# Does an eHealth Intervention Reduce Complications and Healthcare Resources? A mHeart Single-Center Randomized-Controlled Trial

**DOI:** 10.3390/jcdd10020077

**Published:** 2023-02-10

**Authors:** Mar Gomis-Pastor, Sonia Mirabet Perez, Anna De Dios Lopez, Vicenç Brossa Loidi, Laura Lopez Lopez, Rebeca Pelegrin Cruz, Mª Antonia Mangues Bafalluy

**Affiliations:** 1 Digital Health Department and Pharmacy Department, Hospital de la Santa Creu i Sant Pau, IIB Sant Pau, 08025 Barcelona, Spain; 2 Heart Failure and Heart Transplant Unit, Cardiology Department, Hospital de la Santa Creu i Santa Pau and CIBER de Enfermedades Cardiovasculares (CIBER-CV), IIB Sant Pau, 08025 Barcelona, Spain; 3 Pharmacy Department, Hospital de la Santa Creu i Santa Pau, 08025 Barcelona, Spain; 4 Pharmacy Department, Hospital de la Santa Creu i Santa Pau and CIBER de Bioingeniería, Biomateriales y Nanomedicina (CIBER-BBN), 08025 Barcelona, Spain

**Keywords:** heart transplant, eHealth, complications, resource use

## Abstract

(1) Background: In the mHeart trial, we showed that an eHealth intervention, mHeart, improved heart transplant (HTx) recipients’ adherence to immunosuppressive therapy compared with the standard of care. Herein, we present the analysis assessing whether mHeart reduces complication frequency and healthcare resource use, and whether this reduction depends on patients’ adherence. (2) Methods: The mHeart was a single-center randomized-controlled trial (IIBSP-MHE-2014-55) in 134 adult HTx recipients (n = 71 intervention; n = 63 controls). The endpoints were mortality, complications, and resource use during follow-up (mean 1.6 ± 0.6 years). (3) Results: A significantly lower proportion of HTx recipients in mHeart had echocardiographic alteration (2.8% vs. 13.8%; *p* = 0.02), cardiovascular events (0.35% vs. 2.4%; *p* = 0.006), infections (17.2% vs. 56%; *p* = 0.03), and uncontrolled Hba1c (40.8% vs. 59.6%; *p* = 0.03) than controls. In addition, a significantly lower proportion of patients in the intervention needed hospital (32.4% vs. 56.9%; *p* = 0.004) or urgent admissions (16.9% vs. 41.4%; *p* = 0.002) and emergency room visits (50.7% vs. 69.0%; *p* = 0.03). Adherence status (measured by the self-reported SMAQ) influenced only controls regarding hospitalizations and emergency room visits. Differences were not significant on deaths (intervention 4.2% vs. control 9.5%; *p* = 0.4) (4) Conclusions: the mHeart strategy significantly reduced the occurrence of the studied post-transplant complications and the need for medical attention in HTx recipients. Adherence status influenced controls in their need for medical care.

## 1. Introduction

Solid organ transplantation recipients, like many other populations with cardiovascular risk, are vulnerable patients that need long-term and complex management. These patients require lifelong immunosuppressive therapy to avoid graft rejection, which is usually combined with other drugs to treat comorbidities, making recipients take about 10 drugs daily [1]. This therapeutic complexity contributes to the lack of adherence to therapy [2]. Non-adherence is a severe problem for solid organ transplantation patients, especially those who receive heart transplant (HTx). It affects about 40% of HTx recipients [3,4] and can negatively impact the long-term clinical outcomes, namely late acute rejections [5,6,7] and retransplantations, resulting in higher use of resources and medical costs [8]. These deleterious consequences urge us to identify effective interventions in promoting immunosuppressant adherence among solid organ recipients.

In recent years, several studies have indicated that electronic monitoring, pharmacist-led interventions, and cognitive education positively affect immunosuppressant adherence [9]. In addition, mobile app-based health (mHealth) interventions have emerged as up-and-coming tools [10,11,12]. The potential of mHealth technology lies in the great accessibility to smartphones and in their ability to perform several functions simultaneously, such as providing patients with timely reminders to take medications, education about their disease and medication, facilitating telemedicine visits, or transferring patients’ medical information to the healthcare team. Also, mHealth systems offer the opportunity to provide tailored interventions according to individual needs, and deliver interventions to produce cognitive and behavioral change in patients [13].

Based on previous experiences, and following the directions of the International Society for Research on Internet Interventions (ISRII) [13], we developed an mHealth intervention model, the mHeart. The mHeart model combines three theoretical strategies: (1) patient-centered and holistic healthcare models for chronic populations; (2) innovation in clinical practice by including a non-face-to-face care model supported by eHealth; (3) behavioral-based multilevel individually-tailored interventions through eHealth [14]. Together, these strategies aim to improve therapeutic management and care in complex populations such as the HTx recipients through promoting patients’ self-care, resolving patients’ doubts, and facilitating interdisciplinary care according to individual patients’ needs. In terms of adherence, the mHeart is capable of measuring and identifying medication nonadherence and enabling the implementation of behavioral change interventions to improve patient adherence rates. Our single-center randomized-controlled trial (IIBSP-MHE-2014-55) in 134 adult HTx recipients, demonstrated that mHeart improved HTx recipients’ adherence to immunosuppressive therapy compared with the standard of care, with an increase in adherence rate from 46% to 85% (OR = 6.7 [2.9; 15.8]; *p*-value < 0.001), as measured by the validated Simplified Medication Adherence Questionnaire (SMAQ) [15,16].

The main objective of the present post hoc exploratory analysis of the mHeart trial results was to assess whether the addition of the mHeart strategy (intervention group [IG]) compared with the standard of care (control group [CG]) might improve not only adherence but also reduce post-transplant complications and resource use.

## 2. Materials and Methods

### 2.1. Trial Design and Ethical Aspects

This was a post hoc exploratory analysis of the mHeart trial (registered at Clinicaltrials.gov, ID: MHEART: NCT02554578), an open randomized controlled trial (RCT) aimed at comparing the application of the mHeart strategy (IG) with the standard of care (CG) [16]. This post hoc ancillary analysis was focused on assessing whether the intervention reduced the occurrence of complications and the use of resources in adult HTx patients.

As previously detailed [16], the mHeart trial was performed in the heart transplant outpatient setting of the Hospital de la Santa Creu i Sant Pau between July 2015 and 31 December 2018. The trial was approved by the hospital’s Institutional Review Board (IIBSP-MHE-2014-55). Also, all patients were informed of the study’s purposes and procedures, and signed informed consent before their inclusion in the study. For reporting the trial, we adhered to the Consolidated Standards of Reporting Trials of Electronic and Mobile Health Applications and OnLine TeleHealth (CONSORT-EHEALTH) guidelines [17].

### 2.2. Participants

Patients’ randomization, inclusion and exclusion criteria, and sample size calculation are also detailed in Gomis-Pastor et al. [16]. In short, adult HTx recipients were included if they were <1.5 years post-transplant. Conversely, they were excluded if they had a limiting condition that would hinder interviews or the use of the software.

The sample size was based on calculations for the primary outcome of the previous analysis, which was adherence measured with the SMAQ scale. It was thus estimated that a minimum number of 136 patients (including dropouts or losses of at least 10%) was required to achieve a statistical power of 80% to detect a difference of at least 25% in adherence between baseline and 12 months with an alpha level of 0.05. Patients were consecutively enrolled during a baseline visit (T0) and randomly assigned to the IG or the CG in a 1:1 ratio using a random allocation software (Clinapsis^®^) [18].

### 2.3. In-Clinic Visits and Interventions

**Figure 1:**
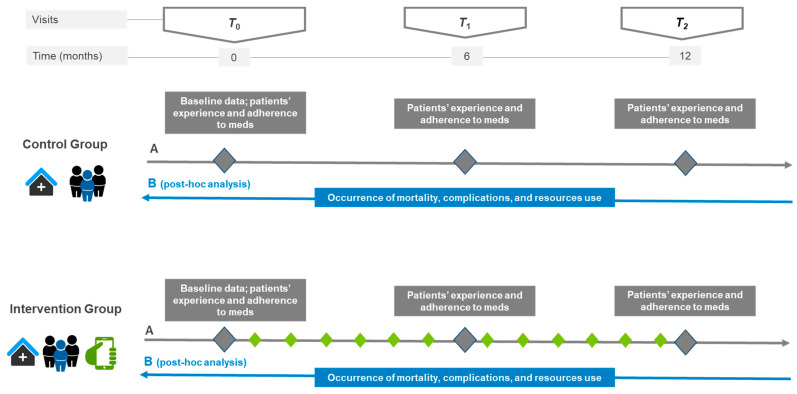
Diagram of the variables collected prospectively during the RCT (A) and retrospectively during the post hoc analysis (B). During the RCT (A), various variables were collected prospectively during the scheduled in-clinic visits: T0 (baseline at study inclusion), T1 (at least 6 months after inclusion), and T2 (at least 12 months after inclusion). The variables assessed during scheduled visits are shown as squares: baseline information, patient experience and medication adherence. For the post hoc analysis (B) the occurrence of mortality, complications, and resource use were collected retrospectively during the study time. Treatments are shown as pictograms, i.e., (i) in-clinic visits at the hospital outpatient department, (ii) multidisciplinary team including the pharmacist, and (iii) the mHeart mobile application for remote interaction with the pharmacist. The diamonds show the scheduled interaction with the clinical pharmacist to perform interventions: gray (during the scheduled in-clinic visits; all patients) and green (using the mHeart tool; intervention group only).

As discussed previously [16] and detailed in Figure 1, the study comprised 3 scheduled in-clinic visits: T0 (baseline at study inclusion), T1 (at least 6 months after inclusion), and T2 (at least 12 months after inclusion) according to the standard of care of our hospital. All patients (CG and IG) received counseling by the pharmacists at T0, T1, and T2 on improving medication self-management through theory-based behavioral interventions [19]. Additionally, patients on the IG were exposed to the mHeart intervention throughout the study to optimize their therapy management. Details on the mHeart clinical use and functionalities have been published elsewhere [15,16]. In addition, descriptions of its development, quality assurance, and feasibility are publicly available [15].

### 2.4. Outcomes

Figure 1 shows the scheduled in-clinic visits and the variables collected during each visit (a) at the previous and (b) at the present analysis. All data collected during the study were recorded electronically in the online database Clinapsis^®^ [18]. In the previous analysis [16], we assessed sociodemographic and baseline clinical data, adherence to medication, and patients’ experience with the medication regimen. The primary outcomes of this post hoc exploratory analysis were the occurrence of mortality, complications, and resource use during the study period.

#### 2.4.1. Health Outcomes

The assessed health outcomes were retrospectively registered from patients’ electronic health records (EHR) at the end of the follow-up period (T2). The outcomes collected per patient were: mortality (yes, no); transplant complications (yes, no; types: cardiac graft vasculopathy, defined as angiographically detectable coronary artery disease classified according to ISHLT nomenclature (i.e., CAV 1–3) [20], revascularization, echocardiographic alteration (considered as the presence of a left ventricular ejection fraction (LVEF) < 55%, or the reduction > 25% of the LVEF that justifies an adjustment of anti-rejection therapy)); cardiovascular events (yes, no; types: cerebrovascular disease, angina, myocardial infarction, heart failure, arteriopathy (defined as peripheral vascular disease with or without symptoms), syncope, atrial fibrillation, requirement for a pacemaker, revascularization, others)); infections (yes, no; and types including any type of bacterial, virus and fungal infections); neoplasm (yes, no; type: cutaneous, pulmonary solid organ, gastrointestinal solid organ, prostate solid organ, lymphoma, others); dialysis (yes, no); and renal function (glomerular filtration, [mL/min]). Also, the following analytical measures were collected from EHR at T0 and T2: LDL cholesterol (mg/dL), hemoglobin A1c (Hba1c; mmol/mol [%]), triglycerides (mg/dL), and high sensitivity troponin T (hs-cTnT; ng/mL) levels. Blood pressure was recorded but not evaluated due to missing data.

#### 2.4.2. Use of Resources

Healthcare resources used per patient refers to all the health centers in the area of Catalonia, and not only to the reference hospital. Data were retrospectively retrieved from the Public data analytics Programme for research and innovation in health (PADRIS) provided by the Agency of Health Quality and Assessment of Catalonia [21]. The types of resources collected per patient were the number of general and urgent hospital admissions, emergency room visits, and general and unscheduled primary care visits.

### 2.5. Analysis

#### 2.5.1. Descriptive Analysis

We estimated absolute and relative frequencies for qualitative variables during the study period (presence of mortality, transplant complications, cardiovascular events, infections, dialysis, renal dysfunction [if glomerular filtration < 60mL/min], high LDL cholesterol [if cholesterol level > 5 mmol/L], high Hba1c [if Hba1c levels > 5.8%], high triglycerides [if triglycerides > 1.70 mmol/L], high troponin [if hs-cTnT level > 13 ng/mL], occurrence of general and urgent hospital admissions, emergency room visits, and general and unscheduled primary care visits). Patients were compared with themselves at the start and at the end of the study period.

#### 2.5.2. Analysis by Objectives

The main objective of this post hoc exploratory analysis was to assess whether the addition of the mHeart strategy (IG) to the standard of care reduces the occurrence of post-transplant complications and the healthcare resources used, namely the need for medical attention, in the follow-up of adult HTx compared to the standard of care (CG) alone in our hospital. To compare the occurrence of complications and healthcare resource use between groups (IG vs. CG), we undertook a contrast analysis by using the Chi-square test and Fisher’s test (variables with frequency < 5%). Also, to assess the influence of the adherence status on the occurrence of complications and healthcare resource use, we first grouped the patients into two adherence groups: adherent and non-adherent patients, measured by the SMAQ [16], and then compared the occurrence of complications and healthcare resource use between these groups through the Chi-square test and Fisher’s test (variables with frequency < 5%).

The data analysis was performed using the IBM-SPSS V25.0 and R versions. For all the statistical tests, the results were considered statistically significant when *p* < 0.05.

## 3. Results

### 3.1. Patient Description

We evaluated health outcomes, and use-of-resource outcomes from the 134 adult HTx patients (n = 71 IG; n = 63 CG) included in the previous mHeart trial report [16]. Patients’ baseline characteristics were previously described [16]. In general, the groups (IG and CG) were well-balanced and not statistically different regarding demographic or clinical information (age at HTx, time from HTx, heart failure etiology, comorbidities, or the immunosuppressive treatment). The patients at baseline (T0) presented several cardiovascular comorbidities; the most frequent were 11 arteriopathies, 8 required pacemakers, 4 revascularizations, 3 cerebrovascular diseases, 3 atrial fibrillations, and 2 syncope myocardia, which represented a mean of 0.33 (SD 0.69) cardiovascular diseases; details are provided in Table 1.

### 3.2. Mortality and Complications during Follow-Up in the Overall Population

The overall population (n = 134) was monitored during a study period of 1.6 years (SD: 0.6). A total of 6.7% (n = 9) patients died during this period. Regarding transplant complications, the most common was cardiac graft vasculopathy, as 54.3% of patients had any degree of CAV according to the ISHLT nomenclature (n = 70/N = 129), including 77.1% CAV 1 (n = 54/N = 70), 7.1% CAV 2 (n = 5/N = 70), and 15.7% CAV 3 (n = 11/N = 70). Of the total number of patients with CAV, 24.3% required stenting (n = 17/N = 70).

A total of 14 cardiovascular events (including 3 cerebrovascular diseases, 2 angina/myocardial infarctions, 2 arteriopathies, 1 atrial fibrillation, 1 pacemaker required, and 3 revascularizations), 22 infections (including 11 respiratory infection, 5 sepsis, 3 cytomegalovirus, 2 influenza, 2 herpes, and 6 others of one single event) and 27 neoplasms (including 14 skin, 5 solid organ, 3 lymphoma, and 5 others of one single event) were recorded during the study. The mean time since the diagnosis of neoplasm from the cardiac transplantation was 9.8 years (SD: 4.0) for skin neoplasm, 11.0 years (SD: 6.0) for solid organ neoplasm, and 16.9 years (SD: 3.2) for lymphoma.

The proportion of patients with renal dysfunction amounted to 48.1% (n = 62/N = 129), and 4.7% (n = 6/N = 129) required dialysis. At the end of the follow-up, almost half of HTx recipients (49.2%; n = 63/N = 128) had high Hba1c levels, whereas 59.7% (n = 74/N = 124) had high hs-cTnT values, and 33.3% (n = 43/N = 129) had triglyceride values exceeding the established threshold.

### 3.3. Differences in Health Outcomes between Patients on Intervention and the Control Group

The comparison between the IG and the CG in terms of health outcomes is shown in Table 2. 

A significantly lower proportion of HTx recipients in mHeart suffered a cardiovascular event (0.35% vs. 2.4; *p* = 0.006) (Table S1) or an infection (17.2% vs. 56.0%; *p* = 0.03) (Table S2) than in the CG during the follow-up. Likewise, at the end of the study, a significantly higher percentage of patients in the CG had higher Hba1c levels (59.6% vs. 40.8%; *p* = 0.03) and higher hs-cTnT levels (51.5% vs. 69.0%; *p* = 0.01) than in the IG (Table S3).

Regarding Hba1c levels, a significantly smaller difference was observed in the CG compared to the IG (*p* = 0.009) when analyzing the differences obtained between the beginning and the end of the study.

The other study variables, namely, mortality, transplant complications such as cardiac graft vasculopathy and revascularization, dialysis requirement, renal dysfunction, and the analytical endpoints LDL cholesterol and triglycerides, did not show statistical differences between the study groups (*p* > 0.05).

### 3.4. Healthcare Resource Use during Follow-Up in the Overall Population

More than 40% (43.4%; n = 56/N = 129) of patients in the general population had undergone hospital admissions and 27.9% required urgent admissions (n = 36/N = 129). Similarly, a large proportion of patients sought emergency care (58.9%; n = 76/N = 129), and this percentage was higher in the case of total (90.7%; n = 117/N = 129) and unscheduled (87.6%; n = 113/N = 129) primary care visits.

### 3.5. Differences in Healthcare Resource Use between Patients in the Intervention and Control Groups

The comparison between the IG and the CG regarding healthcare resource utilization is shown in Table 3.

Alongside the occurrence of the post-transplant complications, a significantly higher percentage of patients in the CG required hospital (56.9% vs. 32.4%; *p* = 0.004), urgent admissions (41.4% vs. 16.9%; *p* = 0.002), and visits to the emergency room (69.0% vs. 50.7%; *p* = 0.03). Also, although the proportion of patients who sought primary care visits was higher in the CG, the differences were not statistically significant.

### 3.6. Differences in Complications and Healthcare Resource Use between Adherent and Non-Adherent Immunosuppressive Treatment

As measured by the SMAQ, 68.2% of the entire population were adherent to the immunosuppressive treatment [16]. The comparison between adherent and non-adherent patients in terms of health outcomes and use of resources is described in Table 4. Accordingly, we observed that a significantly higher proportion of non-adherent patients had cardiovascular events (2.4 vs. 0.85; *p* = 0.04) and infections (31.7% vs. 10.2%; *p* = 0.003) during the follow-up than adherent patients. Likewise, a significantly higher percentage of non-adherent patients required hospital (63.4% vs. 34.1%; *p* = 0.002), urgent admissions (43.9% vs. 20.5%; *p* = 0.005) and visits to the emergency room (78.0% vs. 50.0%; *p* = 0.02).

**Figure 2:**
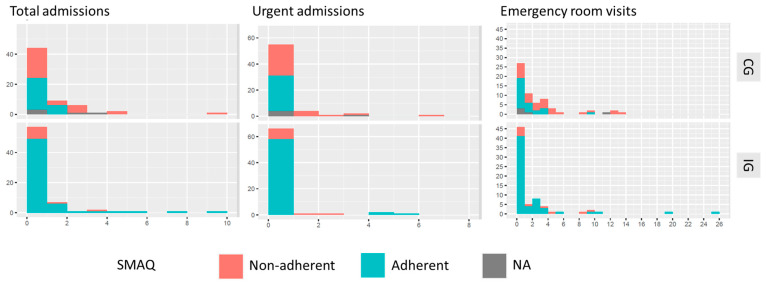
Frequency of admissions and emergency room visits in the control (CG) and intervention (IG) groups and according to the adherence status of patients.

When we assessed the influence of the adherence status on the use of resources according to the study groups (IG or CG), we observed that, among patients in the CG, the adherence status was a factor that significantly decreased both the number of hospitalizations and emergency room visits. However, the impact of the adherence status on the use of resources was not observed in the IG (Figure 2).

## 4. Discussion

Following the positive results from the mHeart trial analysis in improving HTx adherence [16], we set up this post hoc exploratory analysis to assess the hypothesis that the multifaceted theory-based intervention, mHeart, might also reduce post-transplant complications and resource use in addition to improving patients’ adherence. Accordingly, we demonstrated that patients on the mHeart intervention had significantly less occurrence of some cardiovascular events and infections, although we did not observe a difference in mortality. Also, patients on the mHeart showed a lower prevalence of prediabetes and diabetes (high Hba1c levels > 5.8%), a common complication after Htx [22,23]. Lower hs-cTnT levels were observed compared with patients on the standard of care; however this data must be interpreted with caution, since troponins, although they suggest a less likely perioperative cardiac injury [24], are not validated as biomarkers in the monitoring of patients. Moreover, other confounding factors related to different clinical scenarios such as renal disease, age, CAV, and atrial fibrillation, among others, may be involved in the rise of troponin levels [25]. Regarding the resource use, we demonstrated that patients on the mHeart intervention had significantly fewer hospital admissions (both regular and urgent) and emergency rooms visits; after all, it did provide an online communication channel, allowing certain questions to be resolved without an additional presential consult. So far, most of the published studies that have focused on mHealth interventions—including our previous publication [16]—had been limited to showing that these tools promote patients’ commitment to their care and self-reported nonadherence [26,27,28]. However, until now, only a few studies, to our knowledge, have shown the impact of these interventions on mortality or clinical complications such as abnormal laboratory parameters, infections, or use of healthcare resources. In line with our results, an RCT on an mHealth intervention to promote self-management in lung transplantation recipients (N = 201) found increased adherence and less frequent abnormal health indicators in the intervention group, whereas our study groups did not differ in mortality rate [26].

As for the reasons for the differences observed between the groups in our RCT, we hypothesize that an improvement in adherence could drive the reduction of complications, as we observed that non-adherent patients had a higher total number of cardiovascular events and infections. However, other consequences derived from the use of our internet-based multilevel intervention might have influenced these improvements [14]. In particular, mHeart was designed to provide intensive, individually tailored follow-up: patients were contacted at the beginning of the intervention to encourage them in the use of the technology and empower them in their treatment and illness; then, they received periodical personalized feedback through the study to maintain their medical adherence and engagement with their care. These positive reinforcements might be responsible for improving patients’ self-management and ultimately preventing the development of complications. Another possible explanation could be that in the mHearth intervention, warning signals of complications were detected earlier and treated in time to prevent the onset of more severe complications. This early detection ability is possible because our intervention enables health professionals to access patients’ information in a timely manner, provides better communication with patients, and provides better coordination between different healthcare providers.

Another rather novel result in our study was that the patients on the mHeart intervention incurred a lower use of resources such as hospital admissions or visits to the emergency room. These results are in line with those reported in the observational pilot study in lung transplant recipients (N = 56), where it was found that a remote patient-monitoring platform reduced the incidence of hospital readmission (incidence rate ratio [IRR]: 0.38; 95% CI: 0.23–0.63; *p* < 0.001) and days readmitted (IRR: 0.14; 95% CI: 0.05–0.37; *p* < 0.001) [28]. Also, in the observational study assessing telemedicine to follow up the management after liver transplantation (N = 110) [29], the readmission rate within 30 days after discharge was markedly lower in the telemedicine group (*p* = 0.02) [29].

In our case, we hypothesize that this reduced use of resources may correlate with an improvement in recipients’ adherence to overall treatment, along with the availability of a direct communication channel with the medical team, who could answer quickly to the patients’ needs and provide early treatment. These factors combined may have resulted in fewer post-transplant complications, such as infections, which are proven to be the primary cause of hospitalization, even ahead of transplant rejection, for solid organ transplantation recipients [30]. Also, this reduction could be related to the improvement in adherence, as we also noted that a higher percentage of non-adherent patients required hospital admissions and visits to the emergency room. However, in further analysis, we found that adherence status significantly affected the use of resources in those patients in the standard care arm and not those in the arm of the intervention. This distinction could be since the proportion of non-adherent patients was very low in the group of patients in the intervention group, compared with those in the standard of care. Thus, it might not be possible to detect the influence of the adherence status in the intervention group.

This post hoc exploratory analysis is not without limitations. The first is related to the generalizability of the findings, as all patients were recruited from an outpatient center from a single hospital. Hence, we are aware that extrapolating the present findings to other settings should be done with caution, as the standard of care and the characteristics of the population may vary from site to site. The second limitation involves the study design, since it was impossible to conduct a blinded study due to the nature of the intervention. Finally, we are aware that the sample size was estimated based on the main outcome of the previous analysis (statistical power of 80% to detect a difference of at least 25% in adherence but not to detect the difference in event frequency between the two groups). Despite these limitations, we believe our findings are meaningful in our context and might provide a better understanding of the benefits that can be derived from using mHealth approaches for solid organ transplantation recipients.

## 5. Conclusions

In conclusion, this post hoc exploratory analysis is in line with previous findings that demonstrated that the addition of the mHeart strategy to the standard of care of HTx recipients is associated with a significant increase in medication adherence and an improvement in patients’ experience compared with standard care. Here, we demonstrate that this strategy might additionally help to decrease the occurrence of post-transplant complications such as cardiovascular events, infections, or poor analytical endpoints such as glycemic level, among others, and therefore reduce healthcare resource use, especially in terms of the need for medical attention.

## Figures and Tables

**Table 1:** Cardiovascular comorbidities at baseline in the control (CG) and intervention (IG) groups and according to the adherence status of patients.

Num. Cardiovascular Comorbidities	CG Non-Adherent	CG Adherent	IG Non-Adherent	IG Adherent
Without comorbidities	22	22	8	45
1	8	2	2	16
2	0	2	0	0
3	1	0	0	0
4	0	1	0	0

Abbreviations: CG, Control group; IG, intervention group. Information about randomization has been provided in a previous mHeart trial report [16].

**Table 2:** Comparison in the occurrence of mortality, complications, and abnormal laboratory parameters between the control (CG) and intervention (IG) groups.

Variables, n/N (%)	IG	CG	All Patients	*p* Valor
Deceased patients	3/71 (4.2)	6/63 (9.5)	9/134 (6.7)	0.4
Transplant complication				
Cardiac graft vasculopathy	39/71 (54.9)	31/58 (53.4)	70/129 (54.3)	1
Revascularization	7/71 (9.9)	8/58 (13.8)	15/129 (11.6)	0.3
Echocardiographic alteration (LVEF)	2/71 (2.8)	8/58 (13.8)	10/129 (7.8)	0.02
Cardiovascular events *	2/568 ^±^ (0.35)	12/504 ^±^ (2.4)	14/1072 (1.3)	0.006
Infections **	5/29 (17.2)	17/30 (56.0)	22/59 (37.3)	0.03
Dialysis	3/71 (4.2)	3/58 (5.2)	6/129 (4.7)	1
Renal dysfunction (GF < 60 mL/min)	32/71 (45.1)	30/58 (51.7)	62/129 (48.1)	0.3
Analytical endpoints				
High LDL cholesterol	3/71 (4.2)	0/58 (0)	3/129 (2.3)	0.2
High Hba1c	29/71 (40.8)	34/57 (59.6)	63/128 (49.2)	0.03
High triglycerides	22/71 (40.8)	21/58 (36.2)	43/129 (33.3)	0.3
High hs-cTnT	34/66 (51.5)	40/58 (69.0)	74/124 (59.7)	0.01

* Cardiovascular events (cerebrovascular disease, angina, myocardial infarction, heart failure, arteriopathy, syncope, atrial fibrillation, pacemaker required, revascularization, others); ** Infections (sepsis, pneumonia, others), ^±^ % of total cardiovascular events have been referenced to the total number of possible comorbidities (IG: 71 × 8 = 568 and CG: 63 × 8 = 504); Abbreviations: CG, control group; IG, intervention group.

**Table 3:** Comparison in the use of healthcare resources between the control (CG) and intervention (IG) groups.

Variables, n/N (%)	IG	CG	All Patients	*p* Valor
Hospital admissions	23/71 (32.4)	33/58 (56.9)	56/129 (43.4)	0.004
Urgent hospital admissions	12/71 (16.9)	24/58 (41.4)	36/129 (27.9)	0.002
Emergency room visits	36/71 (50.7)	40/58 (69.0)	76/129 (58.9)	0.03
Primary care visits				
Total primary care visits	62/71 (87.3)	55/58 (98.4)	117/129 (90.7)	0.1
Unscheduled primary care visits	59/71 (83.1)	54/58 (93.1)	113/129 (87.6)	0.07

Abbreviations: CG, control group; IG, intervention group.

**Table 4:** Comparison in the occurrence of mortality, complications and abnormal laboratory parameters and use of resources between the adherent and non-adherent patients.

Variables, n/N (%)	Adherent	Non-Adherent	All Patients	*p* Valor
Health outcomes				
Deceased patients	4/88 (4.5)	1/41 (2.4)	5/129 (3.9)	0.7
Transplant complication				
Cardiac graft vasculopathy	52/88 (59.1)	18/41 (43.9)	70/129 (54.3)	0.08
Revascularization	10/88 (11.4)	5/41 (12.2)	15/129 (11.6)	1
Echocardiographic alteration (LVEF)	7/88 (8.0)	3/41 (7.3)	10/129 (7.8)	1
Cardiovascular events *	6/704 ^±^ (0.85)	8/328 ^±^ (2.4)	14/1073 (1.3)	0.04
Infections **	9/88 (10.2)	13/41 (31.7)	22/129 (17.0)	0.003

* Cardiovascular events (cerebrovascular disease, angina, myocardial infarction, heart failure, arteriopathy, syncope, atrial fibrillation, pacemaker required, revascularization, others); ** Infections (sepsis, pneumonia, others), ^±^ % of total cardiovascular events have been referenced to the total number of possible comorbidities (IG: 71 × 8 = 568 and CG: 63 × 8 = 504); Abbreviations: CG, control group; IG, intervention group.

## Data Availability

Data supporting reported results can be found at the database Clinapsis (www.clinapsis.com). An additional dataset was generated during the study in the online Mendeley dataset providing relevant information about the technology for the greater use of the scientific community: Gomis-Pastor M, Mangues MA, Pellicer V. mHeart—mHealthCare Platform Adapted to the Heart Transplant Population—Technical Specifications and Software Source Code. Mendeley Data. doi:10.17632/yf2dgcfmmh.2.

## Supplementary Materials

The following supporting information can be downloaded at: https://www.mdpi.com/article/10.3390/jcdd10020077/s1, Table S1: Supplementary Material. Cardiovascular events described during the study in the control (CG) and intervention (IG) groups; Table S2: Supplementary Material. Infections recorded during the study in the control (CG) and intervention (IG) groups; Table S3: Supplementary Material. Analytical parameters collected during the study in the control (CG) and intervention (IG) groups.
