# Supplementary material for: Does an eHealth Intervention Reduce Complications and Healthcare Resources? A mHeart Single-Center Randomized-Controlled Trial
Source: J Cardiovasc Dev Dis. 2023 Feb 10;10(2):77. doi: 10.3390/jcdd10020077 (PMC9960237; doi:10.3390/jcdd10020077)
Supplement: Supplementary file 1 [file jcdd-10-00077-s001.zip › jcdd-2149361-supplementary.pdf]

Supplementary Material

Table S1. Cardiovascular events described during the study in the control (CG) and intervention (IG) groups.

Cardiovascular event	CG	IG	All participants	P value
Cerebrovascular disease	2	1	3	
Angina / myocardial infarction	2	0	2	
Heart failure	0	0	0	
Arteriopathy	1	1	2	
Syncope	0	0	0	
Atrial fibrillation	1	0	1	
Need pacemaker	1	0	1	
Revascularization	3	0	3	
Others	2	2	4	
**Total n (%)**	**12 (2.4)**	**2 (0.35)**	**14 (1.3)**	**0.006**

Abbreviations: CG, control group; IG, intervention group.

Table S2. Infections recorded during the study in the control (CG) and intervention (IG) groups.

	CG	IG	All participants	P value
Sepsis	4	0	4	
Pneumonia	3	1	4	
Others	10	4	14	
**Total (n; %)**	**17 (56.0%)**	**5 (17.2%)**	**22**	**p=0.03**

Abbreviations: CG, control group; IG, intervention group.

Table S3. Analytical parameters collected during the study in the control (CG) and intervention (IG) groups.

	CG	IG	All participants	P value
High Hba1c (n/N; %)	34/57 (59.6))	29/71 (40.80)	63/128 (49.2)	0.03
High HDL (n/N; %)	5/43 (11.6)	18/55 (32.7)	23/98 (23.5)	0.01
High LDL (n/N; %)	0/43 (0)	1/55 (1.8)	1/98 (1.0)	0.5
High TG (n/N; %)	21/58 (36.2)	22/71 (30.9)	43/129 (33.3)	0.3

Abbreviations: CG, control group; IG, intervention group; Hba1c, glycated haemoglobin A1c; HDL, high-density lipoprotein; LDL, low-density lipoprotein; TG, triglycerides.
